# Cerebellar Neuromodulation Impacts Reading Fluency in Young Adults

**DOI:** 10.1162/nol_a_00124

**Published:** 2024-08-15

**Authors:** Marissa M. Lee, Lauren M. McGrath, Catherine J. Stoodley

**Affiliations:** Center for Applied Brain and Cognitive Sciences, Tufts University, Medford, MA, USA; Department of Neuroscience, American University, Washington, DC, USA; Department of Psychology, University of Denver, Denver, CO, USA

**Keywords:** cerebellum, neuromodulation, processing speed, reading accuracy, reading fluency, transcranial direct current stimulation

## Abstract

The cerebellum is traditionally associated with the control of coordinated movement, but ample evidence suggests that the cerebellum also supports cognitive processing. Consistent with this, right-lateralized posterolateral cerebellar regions are engaged during a range of reading and reading-related tasks, but the specific role of the cerebellum during reading tasks is not clear. Based on the cerebellar contribution to automatizing movement, it has been hypothesized that the cerebellum is specifically involved in rapid, fluent reading. We aimed to determine whether the right posterolateral cerebellum is a specific modulator of reading fluency or whether cerebellar modulation is broader, also impacting reading accuracy, rapid automatized naming, and general processing speed. To do this, we examined the effect of transcranial direct current stimulation (tDCS) targeting the right posterolateral cerebellum (lobules VI/VII) on single-word reading fluency, reading accuracy, rapid automatized naming, and processing speed. Young adults with typical reading development (*n* = 25; 15 female sex assigned at birth, 10 male sex assigned at birth, aged 18–28 years [*M* = 19.92 ± 2.04 years]) completed the reading and cognitive measures after 20 min of 2 mA anodal (excitatory), cathodal (inhibitory), or sham tDCS in a within-subjects design. Linear mixed effects models indicated that cathodal tDCS decreased single-word reading fluency scores (*d* = −0.36, *p* < 0.05) but did not significantly affect single-word reading accuracy, rapid automatized naming, or general processing speed measures. Our results suggest that the right posterolateral cerebellum is involved in reading fluency, consistent with a broader role of the cerebellum in fast, fluent cognition.

## INTRODUCTION

### Reading and the Cerebellum

#### Typical reading development

**Figure 1.**
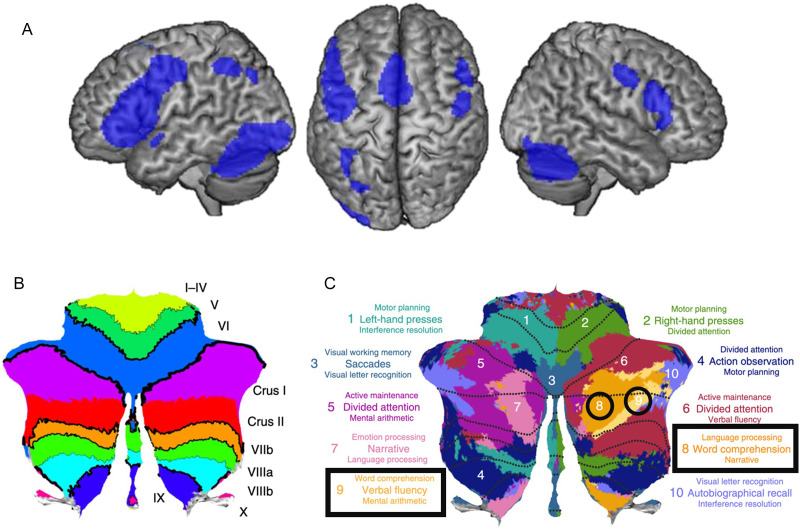
(A) Reading network in adults with typical reading development; adapted from Martin et al. (2015). (B) Cerebellar lobules labeled on a flatmap. (C) Functional parcellation of the cerebellum (colors) and anatomical lobules (dotted lines); adapted with permission from King et al. (2019).

The traditional reading network is comprised primarily of left-lateralized cortical regions including the inferior frontal gyrus (IFG), temporoparietal cortex, and occipitotemporal cortex (e.g., Kearns et al., 2019; Murphy et al., 2019), but there is also strong evidence for a role of the cerebellum in reading (for review, see Alvarez & Fiez, 2018; for meta-analysis, see Martin et al., 2015). Functional mapping of the cerebellum indicates right posterolateral regions (e.g., lobule VII, including Crus I and II; see Figure 1) are associated with multiple aspects of reading, such as visual letter recognition and word comprehension (King et al., 2019), as well as lexical and sublexical processing (for reviews, see Stoodley & Stein, 2011; Vlachos et al., 2007). A meta-analysis of functional magnetic resonance imaging (fMRI) studies of adult readers showed convergence of reading-related activation in the right posterolateral cerebellum together with canonical cortical reading network regions (Martin et al., 2015). More recently, D’Mello et al. (2020) reported that right posterolateral cerebellar regions (e.g., Crus II, lobule VIIb, lobule VIII) were associated with reading in adolescents and adults and that activity levels within these regions varied depending on factors such as sentence plausibility and presentation rate. Similar cerebellar regions were also engaged in a sample of children first learning to read (Li et al., 2021). As the children’s reading ability improved and brain networks matured over 1–1.5 years, cerebellar activation shifted from left lobule VII to right lobule VII, mimicking adult patterns of activity.

In line with the functional findings, structural imaging has also shown that gray matter (GM) volume in a large bilateral cluster encompassing cerebellar Crus II, lobule VIIB, and lobule VIII was positively associated with single-word reading in a large sample of children and adolescents (Moore et al., 2017). He et al. (2013) reported similar findings: GM volume within left Crus II and right lobule VIIb was positively associated with vocabulary, a correlate of reading skills, in Chinese readers. These imaging findings indicate that the cerebellum is functionally engaged during reading tasks and that cerebellar GM volume correlates with reading-related measures.

While both functional and structural imaging studies support a role of the cerebellum in reading, imaging analyses often focus only on the cerebrum or cortical regions of interest that have been associated with the traditional reading network. Further, not all imaging studies of reading report significant cerebellar activation (e.g., Ashburn et al., 2020), suggesting that cerebellar engagement during reading may be task-dependent. Therefore, the specific contribution of the cerebellum to reading has yet to be established.

#### Developmental dyslexia

Developmental dyslexia (DD) is diagnosed when an individual displays unexpected difficulties in word decoding skills compared to age-matched peers despite adequate access to literacy education (Shaywitz & Shaywitz, 2005; Shaywitz et al., 2021). Prevalence estimates of DD indicate that it is one of the most common learning disabilities with approximately 10%–20% of individuals meeting diagnostic criteria (Shaywitz et al., 2021). Critically, reading interventions in DD populations often successfully improved reading accuracy skills, but it has proven more difficult to improve reading fluency skills (Alexander & Slinger-Constant, 2004; Norton & Wolf, 2012).

Neural differences associated with DD converge primarily on the cortical regions of the reading network, namely the left IFG, temporoparietal cortex, and occipitotemporal cortex. A series of meta-analyses have indicated hypoactivation of these regions in individuals diagnosed with DD compared to typical readers (Richlan, 2012; Richlan et al., 2009, 2011; Zhang & Peng, 2022), even across orthographic depths (Martin et al., 2016) and different writing systems (Yan et al., 2021).

Although there is a focus on the left-lateralized reading network, functional neuroimaging studies also suggest that individuals with DD recruit brain regions more bilaterally than individuals with typical reading development, including the reading network’s right hemisphere homologues and additional right and left hemisphere regions (Pollack et al., 2015). Meta-analyses of anatomical differences between individuals with DD and typical readers report bilateral findings in DD, with GM increases and decreases in both hemispheres (Richlan et al., 2013; Yan et al., 2021).

There is also evidence of cerebellar differences in DD compared to typical readers. A meta-analysis performed by Yan et al. (2021) revealed hypoactivation in left cerebellar lobule VI and Crus I and hyperactivation in right lobule VI in DD. Furthermore, there were also structural differences in the DD sample, with increased GM volume in left Crus I and decreased GM volume in right lobule VI relative to typical readers. Cerebellar structural and functional differences in DD converge with the cerebellar regions engaged during reading tasks in typical readers.

That said, cerebellar differences are not consistently reported in functional imaging meta-analyses of DD. The reading tasks presented during fMRI studies of DD are usually untimed tasks focused on phonological awareness or decoding (e.g., rhyme judgement, untimed reading of single words, untimed reading of pseudowords), which may not optimally engage the cerebellum. Yan et al. (2021) was the only meta-analysis to report cerebellar functional differences between individuals with DD and typical readers, possibly because their analysis included studies tapping a wider variety of cognitive tasks (e.g., picture n-back, sound discrimination, motion detection). This suggests that cerebellar differences in DD may be associated more with domain-general cognitive skills rather than specific untimed reading measures.

#### The cerebellum, skill automatization, and reading fluency

If the role of the cerebellum is more domain-general, then how is the cerebellum involved in reading? The answer may lie in the cerebellum’s role in skill learning and automatization. While phonological deficits have been extensively documented in DD, these are not the only cognitive differences evident in DD (Pennington, 2006; Snowling et al., 2020). Individuals diagnosed with DD also experience challenges with reading fluency (Snowling et al., 2020). Fluent reading is achieved when reading becomes an automatic skill. The cerebellum has been consistently associated with both motor and cognitive skill automatization (Schmahmann et al., 2019; Sokolov et al., 2017), and it has been suggested that cerebellar disruption in DD could impair the automatization of the skills necessary for fast, fluent reading (Nicolson & Fawcett, 2019; Norton et al., 2014).

Functional imaging studies support a cerebellar role in reading fluency, including right posterolateral cerebellar regions such as lobule VI. Functional connectivity between right lobule VI and the left supramarginal gyrus, part of the temporoparietal reading network hub, was associated with reading fluency as opposed to phonological awareness in typical readers (Ang et al., 2020). Norton et al. (2014) observed a relationship between worse Rapid Automatized Naming (RAN) scores and decreased right lobule VI activation in a sample of children with DD (defined by the double-deficit model), a pattern that was not evident in the Elision and Blending Words phonology tasks. This pattern is notable because RAN is one of the strongest cognitive correlates of reading fluency measures (Norton & Wolf, 2012). Cummine et al. (2015) compared brain activation patterns in response to alphanumeric RAN tasks and rapidly reading words and nonwords. They found that all RAN measures engaged cerebellar vermal VI extending into right lobule VI, and that rapidly reading letters and nonwords both engaged right Crus I. Additional cerebellar regions also appear to be associated with reading fluency when manipulating the presentation speed of letters and sentences and therefore driving the rate of reading. In readers with typical development, faster presentation rate was associated with increased activity in left lobule VII (Crus I and II; Christodoulou et al., 2014) and bilateral Crus I and II (Benjamin & Gaab, 2012). In a DD sample, increasing the presentation rate resulted in increased activity in cerebellar vermal IX (Christodoulou et al., 2014).

Reading fluency has also been associated with processing speed, defined as the speed at which one can accurately complete a task with a high cognitive load (Jacobson et al., 2011; Kail & Salthouse, 1994). One explanation for slower RAN performance in DD is that it may be at least partially attributed to underlying processing speed weaknesses (Catts et al., 2002; McGrath et al., 2011; Norton & Wolf, 2012; Wolf & Bowers, 1999). In a latent analysis of cognitive skills associated with word decoding, RAN and processing speed showed a very high correlation with each other of *r* = 0.74 in a large, community-based sample of youth (McGrath et al., 2011). Behaviorally, there is contradictory evidence as to whether the processing speed weaknesses seen in DD are restricted to tasks most closely associated with reading (e.g., alphanumeric RAN measures) or whether there is slower processing speed across a range of tasks (McGrath et al., 2011; Norton & Wolf, 2012). Notably, the cerebellum is a neural region of overlap associated with both reading fluency and general processing speed measures (Eckert et al., 2010; Genova et al., 2009; Razlighi et al., 2017; Wong et al., 2021). This raises the question of whether the posterolateral cerebellum can be more accurately described as being a neural correlate of general processing speed, making performance fast and efficient across a range of cognitive tasks, which manifests as reading fluency in the reading domain.

### Modulation of the Cerebellum with Transcranial Direct Current Stimulation

How can we determine the specific impact of a brain region on behavior? One approach is to transiently alter the activity of that region using neuromodulation. Transcranial direct current stimulation (tDCS) is a noninvasive method that allows for the manipulation of neural activity by applying a small amount of direct current to the scalp. The current alters the membrane potential of the underlying brain tissue, either in an excitatory (anodal stimulation) or an inhibitory (cathodal stimulation) manner. Sham tDCS can be used as a control condition, where the current is briefly ramped up and then ramped down so individuals will experience the initial sensations associated with active tDCS, but not enough current to impact the underlying tissue. The amount of current used in tDCS studies commonly ranges from 1–2 mA and is applied for approximately 20 min at a time (Ferrucci et al., 2015), producing effects that last ∼45–60 min. TDCS has been used in hundreds of studies with no significant adverse effects (Bikson et al., 2016). Typical side effects include itching, tingling, or burning at the electrode sites, but these tend to be mild in intensity (Kessler et al., 2012).

Cancer and Antonietti (2018) reviewed tDCS studies of reading in individuals with DD and typical readers. While a variety of protocols have been used (e.g., varying in the amount of current, location of electrodes, reading subprocess measured), the most common configuration was anodal stimulation of the left temporoparietal cortex. This review highlighted different effects across age groups and in DD cohorts compared to typically developing readers. Across modulation sites, tDCS was found to have the greatest impact on real word reading in adults and nonword or low-frequency word reading in children and adolescents. Anodal tDCS targeting the left temporoparietal area increased word reading efficiency of low-frequency and nonwords in those diagnosed with DD, though this relationship was not reported consistently in typical readers.

It is also possible to perform tDCS on the cerebellum. For reading and language tasks, usually the right hemisphere is targeted (e.g., D’Mello et al., 2017) because it communicates contralaterally with the cortex (Kelly & Strick, 2003) and, more specifically, with the left-lateralized reading network (Alvarez & Fiez, 2018). Also, engagement of right posterolateral cerebellar regions has been specifically associated with a range of reading-related tasks (Cummine et al., 2015; King et al., 2019; Stoodley et al., 2012). Generally, anodal stimulation of the cerebellum is thought to lead to improvements in performance whereas cathodal stimulation disrupts task performance, though there are some inconsistencies between studies (van Dun et al., 2016). Anodal tDCS targeting the right posterolateral cerebellum has been shown to increase phonemic fluency in typically developing adults (Turkeltaub et al., 2016). While D’Mello et al. (2017) did not find evidence that anodal stimulation to the right posterolateral cerebellum affected performance on a sentence prediction task, they did report increased functional connectivity between right posterolateral cerebellum and cortical regions, specifically areas implicated in the reading network. In contrast with anodal tDCS, cathodal stimulation of the right cerebellar hemisphere decreased verbal working memory (Boehringer et al., 2013) and verb generation (Spielmann et al., 2017), though there was no effect on reading speed (Boehringer et al., 2013). That said, cathodal tDCS has also been shown to improve working memory and verb generation (Pope & Miall, 2012), and Turkeltaub et al. (2016) reported a trend-level (*p* = 0.057) increase in verbal fluency following cerebellar cathodal tDCS. Therefore, the polarity-specific effects of cerebellar tDCS on cognitive task performance have been inconsistent and preclude strong hypotheses about the anticipated direction of behavioral change.

### Current Study

We aimed to evaluate the role of the cerebellum in reading through manipulating cerebellar activity with tDCS. We included untimed single-word reading accuracy tasks, timed single-word and nonword reading fluency tasks, RAN measures, and general processing speed tasks not requiring reading. Our goals were: first, to determine whether the right posterolateral cerebellum specifically supports reading fluency (vs. accuracy); and second, to evaluate whether the right posterolateral cerebellum plays a more domain-general role (e.g., cerebellar tDCS impacts all timed tasks) or a domain-specific role (e.g., cerebellar tDCS impacts only reading-specific tasks) in reading.

We hypothesized that modulation of right posterolateral cerebellum would affect timed reading tasks but not untimed reading tasks, indicating that the cerebellum is most important for reading fluency in adult readers. While previous cerebellar tDCS studies have had mixed findings regarding the effect of polarity on behavioral performance, we tentatively predicted that anodal stimulation would improve reading speed, whereas cathodal stimulation would disrupt reading speed. We did not have an a priori prediction about whether the cerebellar role in reading speed could be explained by a more general role in processing speed, but if tDCS targeting the right posterolateral cerebellum affects reading fluency and RAN scores but not general processing speed, it would indicate a domain-specific role for the right posterolateral cerebellum in reading fluency, possibly via modulation of the connections between the right posterolateral cerebellum and the left-hemisphere cortical reading network.

## MATERIALS AND METHODS

### Participants

Participants included 27 right-handed native English-speaking adults 18–28 years old with no history of learning, neurological, psychiatric, developmental, or language disorders and who were not currently taking any medications that may influence the central nervous system. Participants also reported no contraindications for tDCS (e.g., no metallic fragments in the head, no implanted electrical devices, no history of head trauma, epilepsy, or brain-related injuries, not currently pregnant when applicable). Participants were recruited via advertisements at the university and other local institutions. All participants gave written informed consent and were compensated for their time. All procedures were approved through the American University Institutional Review Board.

Two participants were removed from the final sample—one because of multiple low behavioral scores that indicated reading difficulties and one because of a National Institutes of Health (NIH) Toolbox Cognition Battery composite score greater than 2 *SD* below average. The final participant sample, described further in Table 1, included 25 typical readers (15 females, 10 males, 18–28 yr old, *M* = 19.92 yr, *SD* = 2.04 yr).

**Table 1.** Participant demographics

	*N*		
Total	25		
	Mean	*SD*	Range
Age (years)	19.92	2.04	18–28
Education (years)	14.32	1.22	13–17
Mother’s education (years)	17.16	2.15	13–21
	*N*	%	
Sex			
Male	10	40%	
Female	15	60%	
Race			
White	16	64%	
Black or African American	1	4%	
Asian	3	12%	
Multiracial*	1	4%	
Self-described	4	16%	
Ethnicity			
Not Hispanic or Latino/a/x	20	80%	
Hispanic or Latino/a/x	4	16%	
Declined to respond	1	4%	

*Note*. * Multiracial was assigned if the participant selected more than one given option.

### Behavioral Measures

All participants completed a series of behavioral questionnaires, cognitive assessments, reading tasks, and processing speed tasks. Reading measures included both timed and untimed tasks to measure single-word and nonword reading fluency and accuracy, respectively.

The National Institute for Children’s Health Quality (NICHQ) Vanderbilt Assessment Scale (VAS; Wolraich et al., 2003) was used to evaluate ADHD-related symptoms. The full assessment scale was administered (47 items), but this study used the nine symptoms of inattention and nine symptoms of hyperactivity/impulsivity that align with *Diagnostic and Statistical Manual of Mental Disorders* (5th ed., text rev.; DSM-5-TR; American Psychiatric Association, 2022) symptoms of ADHD. Each symptom is rated based on how often each behavior occurred in the past six months.

The Dimensional Change Card Sort, Flanker Inhibitory Control and Attention Test, List Sorting Working Memory, Picture Sequence Memory Test, and Picture Vocabulary Test subtests from the NIH Toolbox Cognition Battery (Weintraub et al., 2013) were administered and combined to form a composite IQ estimate. The NIH Toolbox Cognition Battery was administered via iPad. Each task was presented consecutively in the same order for each participant and was automatically scored.

Reading tasks were chosen from timed and untimed standardized measures. Subtests from Woodcock Reading Mastery Tests (WRMT-III; Woodcock, 2011) measured single-word reading accuracy (Word ID), single nonword reading accuracy (Word Attack), and a predictor of reading fluency (RAN letters and digits). The Word Reading subtest from the Wide Range Achievement Test (WRAT5; Wilkinson & Robertson, 2017) was also used to measure single-word reading accuracy. Measures from the Test of Word Reading Efficiency (TOWRE-2; Torgesen et al., 2012) measured single-word reading fluency (Sight Word Efficiency; SWE) and single-nonword reading fluency (Phonemic Decoding Efficiency; PDE). General processing speed was measured with the Coding and Symbol Search subtests of the Wechsler Adult Intelligence Scale (WAIS-IV; Wechsler, 2008). See Table 2.

**Table 2.** Reading and processing speed tasks

Task	Relative to tDCS application	Description
Reading Fluency (Timed)		
TOWRE-2 SWE	Pre- and post-tDCS	Number of real single words from a given list that are accurately read in 45 s
TOWRE-2 PDE	Pre- and post-tDCS	Number of single nonwords from a given list that are accurately read in 45 s
Reading Accuracy (Untimed)		
WRMT-III Word ID	Pre-tDCS	Number of real single words that are accurately read from a list
WRMT-III Word Attack	Pre-tDCS	Number of single nonwords that are accurately read from a list
WRAT-5 Word Reading	Post-tDCS	Number of real single words that are accurately read from a list
Reading-Related Processing Speed (Timed)		
RAN-Letters	Pre- and post-tDCS	Amount of time it takes to read through a matrix of letters
RAN-Digits	Pre- and post-tDCS	Amount of time it takes to read through a matrix of numbers
General Processing Speed (Timed)		
Coding	Post-tDCS	Number of correctly copied symbols paired with a number in 2 min
Symbol Search	Post-tDCS	Number of correctly identified target symbols in 2 min

Reading comprehension worksheets were administered during tDCS. They were obtained online from an open access educational website with activities for grades K–12. A total of nine different passages rated at a sixth-grade level were given across the three sessions, three for each session. Participants were instructed to read the passages and answer the corresponding comprehension questions at their own pace. They did not need to finish all three reading comprehension worksheets during the 20 min of tDCS administration. Answers were not scored. The purpose of the reading comprehension worksheets was to engage the reading network at a level that would not be too mentally taxing. While the cerebellum was the targeted brain region for neuromodulation, stimulation likely impacts additional regions with which the cerebellum is functionally connected (e.g., Rice et al., 2021), such as the cortical reading network. Engaging the reading network could make these regions preferentially sensitive to tDCS neuromodulation (Bikson & Rahman, 2013), possibly increasing the effect of tDCS.

### Study Design

After consenting to participation and reviewing COVID-19 symptoms and protocols, participants completed the NICHQ VAS and the tDCS safety screening questionnaire. Each participant completed three study sessions one week apart, with anodal, cathodal, and sham tDCS administered in a within-participants design. The order of stimulation type was counterbalanced across participants. At the start of every session, individuals whose sex assigned at birth was female were asked to take a pregnancy test as tDCS safety has not been tested during pregnancy.

During session 1, participants completed the NIH Toolbox Cognition Battery, Word ID, Word Attack, RAN-letters, RAN-digits, SWE, and PDE measures to characterize general cognitive and reading ability prior to tDCS (pre-tDCS). Participants then underwent 20 min of cerebellar tDCS (more details in Neuromodulation Protocol). During tDCS, participants completed three reading comprehension worksheets rated at a sixth-grade level to engage reading-related brain networks. After tDCS, participants completed a second set of SWE and PDE measures using alternate forms, and a second set of RAN-letters and RAN-digits, Word Reading, Symbol Search, and Coding (no alternate forms available).

Sessions 2 and 3 began with 20 min of tDCS while participants completed reading comprehension worksheets. After tDCS, they completed the same post-tDCS measures as session 1 using separate forms when available (see details below).

At the end of every session, participants rated side effects both during and after tDCS with a 26-item questionnaire, including tingling, itching, burning, attention, fatigue, and pain (see Kessler et al., 2012). Participants rated the extent to which they experienced these symptoms on a scale of 0 (*not at all*) to 10 (*greatest imaginable*).

First, the measures themselves were counterbalanced so reading fluency, reading accuracy, rapid naming, and processing speed measures were administered in a different order between individuals and between sessions. Then, if alternate forms were available, they were also counterbalanced between participant and session. SWE and PDE forms (A, B, C, D) were administered once each (pre-tDCS session 1, post-tDCS session 1, post-tDCS session 2, post-tDCS session 3). WRAT5 Word Reading forms (blue, green) were given in a pseudorandomized order, either blue/green/blue or green/blue/green (post-tDCS in sessions 1, 2, and 3). The counterbalanced order was computed via Latin squares in R (Version 4.1.2; R Core Team, 2021) and then manually inspected to determine that each measure and form (when applicable) were equally as likely to be placed in any order position.

**Figure 2.**
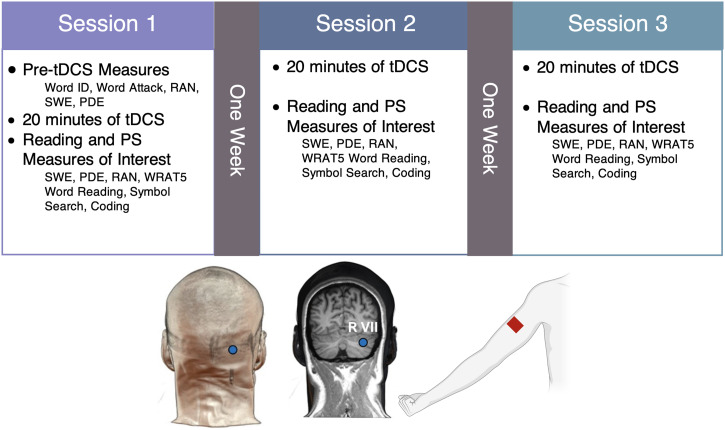
Overview of the three-week experimental design and tDCS montage. Stimulation type and task order/forms were counterbalanced across sessions. TDCS montage shows target electrode placement on the skull (1 cm below and 4 cm to the right of the inion), targeted cerebellar region (right posterolateral cerebellum, overlapping with lobules VI and VII), and reference electrode placement (right upper arm). tDCS = transcranial direct current stimulation; PS = processing speed; SWE = Sight Word Efficiency; PDE = Phonemic Decoding Efficiency; RAN = Rapid Automatized Naming; WRAT5 = Wide Range Achievement Test (5th ed.).

A summary of the study design can be found in Figure 2 and Table 2.

### Neuromodulation Protocol

Cerebellar neuromodulation was performed with a NeuroConn DC Stimulator Plus (Jali Medical, Inc, Waltham, MA). Participants received 2 mA of current targeting the right posterolateral cerebellum (overlapping with lobules VI and VII) for 20 min using a 5 × 5 cm saline-soaked sponge electrode. The target electrode was placed 4 cm to the right and 1 cm below the inion (Ferrucci et al., 2015; Figure 2). The return electrode was placed on the ipsilateral (right) upper arm. Each participant received anodal, cathodal, and sham conditions in a within-subject design with order of tDCS polarity counterbalanced over the three study sessions.

The tDCS montage is very similar to that used in Rice et al. (2021), which also targeted the right posterolateral cerebellum, except that Rice et al. placed the reference electrode on the ipsilateral clavicle. Estimated electrical field maps indicated that the current primarily impacts the right cerebellar hemisphere with this montage (see Rice et al., 2021).

### Data Analyses

Data normality was assessed through a combination of visually inspecting density, quantile–quantile and residual plots, and Shapiro-Wilk’s test of normality (R *graphics* and *stat* packages). All data were normally distributed. Possible outliers were assessed with the outlier test from the R *car* package. No outliers were detected.

Analyses focused on evaluating reading fluency, reading accuracy, RAN, and processing speed differences between tDCS conditions (anodal, cathodal, sham). Post-anodal and post-cathodal performance was compared to post-sham performance in a within-subjects analysis. During sham tDCS, not enough current is applied to the individual to cause any physiological or behavioral changes, therefore it can be considered a control condition. Separate models assessed the effects of cerebellar tDCS on individual single-word and nonword reading fluency scores, single-word reading accuracy, RAN, and processing speed scores. Performance on the task of interest was the dependent variable, with tDCS condition (anodal, cathodal, sham), and session (1, 2, 3) as independent variables. Session was included to account for potential learning effects across the three study sessions. An interaction effect between tDCS type and session was also included in the model. Linear mixed effects (LME) models (R *lme4* package) were used to account for the random effect of participant.

$$\text{Fluency}/\text{Accuracy}/\text{RAN}/\text{PS}∼\text{tDCS}+\text{session}+\text{tDCS}*\text{session}+\left(1|\text{Participant}\right)$$

The model was reduced by removing the interaction effect if the interaction did not reach significance (*p* < 0.05). The final models were evaluated first at *p*_uncorrected_ < 0.05. To correct for multiple comparisons, we also evaluated all final models at *p*_corrected_ < 0.05 using the Benjamini-Hochberg procedure (Benjamini & Hochberg, 1995). We report the uncorrected *p* values, and if significance was met, we also report the corrected *p* values. A full list of corrected *p* values can be found in Table S2, available in the Supporting Information at https://doi.org/10.1162/nol_a_00124. Effect sizes were calculated based on the following equation from Brysbaert and Stevens (2018):

$$d=\left(\text{difference}\;\text{between}\;\text{means}\right)/\text{sqrt}(\text{variance}\;{\text{intercept}}_{\text{participant}}+{\text{variance}}_{\text{residual}})$$

Effect sizes are reported for significant *p* values. A full list of effect sizes can be found in Table S3.

## RESULTS

All participants had NIH Toolbox Cognition Battery Composite scores within the typical range (>85 standard score). NICHQ VAS results indicated that no participants had clinically elevated ADHD symptomatology (Table 3).

**Table 3.** Participant characteristics

	Mean	*SD*	Range
NIH Cognitive Toolbox composite standard score	104	12.2	85–131
NICHQ VAS			
Number of inattentive symptoms**	0.3	0.6	0–2
Number of hyperactive/impulsive symptoms**	0.4	1.0	0–4
Word ID standard score	106.4	7.6	92–118
Word Attack standard score	104.4	12.3	83–121

*Note*. ** Maximum number of symptoms was nine. DSM-5 (American Psychiatric Association, 2022) ADHD cut-offs suggest six or more symptoms in either domain meet symptom criteria for ADHD. NIH = National Institutes of Health; NICHQ VAS = National Institute for Children’s Health Quality Vanderbilt Assessment Scale.

### TDCS Side Effects and Tolerability

No significant adverse tDCS side effects were reported. Most side effect ratings during and after tDCS were very low in intensity (e.g., <1 on a scale of 1–10). The highest ratings were given to side effects during tDCS, namely itching (1.54 ± 2.13 during sham tDCS, 3.64 ± 2.34 during anodal tDCS, 1.78 ± 2.03 during cathodal tDCS), burning (1.02 ± 1.98 during sham tDCS, 0.94 ± 1.61 during anodal tDCS, 1.40 ± 2.12 during cathodal tDCS), and tingling sensations (1.76 ± 1.71 during sham tDCS, 2.18 ± 1.67 during anodal tDCS, 2.54 ± 1.97 during cathodal tDCS; Table S1). Only itching during tDCS was significantly different between stimulation types (*F*(2, 48) = 11.33, *p* < 0.001). A Tukey post hoc test showed that anodal stimulation resulted in higher itchiness ratings compared to sham (*p* = 0.002) and cathodal stimulation (*p* = 0.003).

### Reading, RAN, and Processing Speed Scores

As expected, reading, RAN, and processing speed scores fell within the typical range (Table 4).

**Table 4.** Reading, RAN, and processing speed scores

**Task**	Sham	Anodal	Cathodal	**Relative Difference**	
				Sham-Anodal	Sham-Cathodal
**Timed single-word reading**					
PDE	108.4 ± 8.3 [91–127]	110.5 ± 8.8 [100–130]	109.8 ± 8.9 [100–127]	2.0 ± 7.8 [−17–23]	1.4 ± 8.8 [−19–25]
SWE	116.1 ± 12.6 [91–130]	114.3 ± 8.6 [101–130]	112.1 ± 11.4* [91–130]	−1.8 ± 8.3 [−17–15]	−4.0 ± 9.6 [−24–13]
**Untimed single-word reading**					
Word Reading	113.3 ± 6.4 [100–125]	115.4 ± 9.7 [98–135]	114.5 ± 7.6 [100–126]	2.1 ± 7.5 [−9–10]	1.2 ± 6.0 [−12–13]
**Rapid Automatized Naming (RAN)**					
RAN	70.5 ± 9.1 [56–80]	71.3 ± 8.7 [52–80]	69.9 ± 10.8 [51–80]	0.8 ± 4.9 [−7–10]	−0.6 ± 3.8 [−7–8]
**Processing speed**					
Symbol Search	13.0 ± 3.4 [6–19]	13.7 ± 3.9 [6–19]	12.6 ± 3.3 [7–18]	0.7 ± 3.2 [−5–6]	−0.4 ± 3.1 [−7–5]
Coding	13.4 ± 3.4 [8–19]	13.4 ± 3.6 [7–19]	13.2 ± 3.2 [7–19]	−0.1 ± 1.4 [−3–2]	−0.2 ± 1.7 [−3–4]

*Note*. Scores are reported as *M* ± *SD* [min-max]; PDE, SWE, and Word Reading are reported as standard scores with a mean of 100 ± 15. Coding and Symbol Search are reported as scaled scores with a mean of 10 ± 3. RAN scores are reported as raw scores with a maximum of 80.

* Significant difference in scores compared to sham stimulation, *p*_uncorrected_ < 0.05.

### Correlations Between Reading Fluency, RAN, and Processing Speed Measures

**Figure 3.**
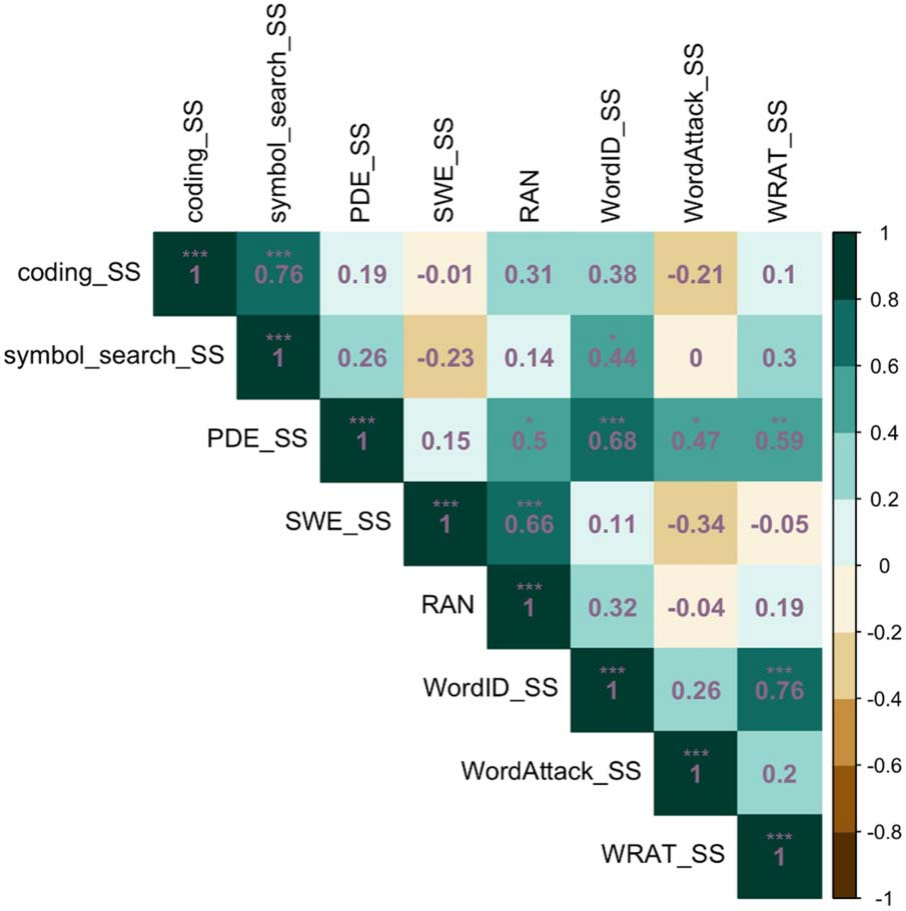
Correlation matrix for all tasks in the absence of neuromodulation. SS = standard or scaled score. * *p* < 0.05, ** *p* < 0.01, *** *p* < 0.001.

To guide our understanding of the relationship(s) between reading fluency, RAN, and processing speed measures, correlation analyses were conducted on data collected pre-tDCS (Word Attack and Word ID measures) and after sham tDCS. There were no significant correlations between reading fluency and general processing speed scores (*r* = −0.01–0.26, *p* > 0.05; Figure 3). There were significant correlations between SWE and RAN (*r* = 0.66, *p* < 0.001) and PDE and RAN (*r* = 0.5, *p* = 0.02), consistent with the previously described relationship between RAN and reading fluency (e.g., Norton & Wolf, 2012). Interestingly, there was a significant correlation between pre-tDCS Word ID reading accuracy scores and Symbol Search processing speed scores (*r* = 0.44, *p* = 0.03); for Coding the relationship was not statistically significant (*r* = 0.38, *p* > 0.05).

### Impact of Cerebellar tDCS on Reading and Processing Speed Measures

#### Effect of cerebellar tDCS on reading fluency and RAN

Backward stepwise regression removed the nonsignificant interaction term (tDCS × Session) in the reading fluency and RAN models, resulting in reduced models with only the effects of tDCS and session.

**Figure 4.**
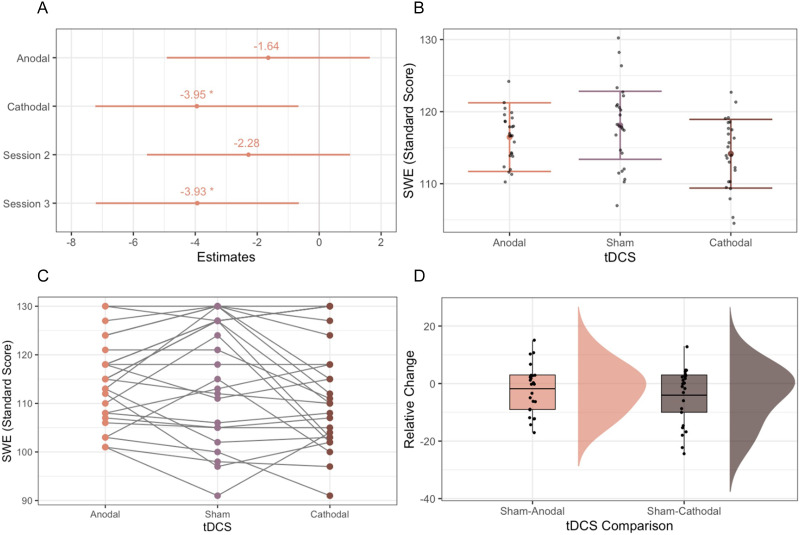
(A) Linear mixed effects model standard beta estimates for SWE. The sham tDCS condition was used as a reference for the Anodal and Cathodal conditions. Session 1 was used as a reference for Sessions 2 and 3. (B) SWE scores for each stimulation type, controlling for effects of session. (C) Changes in SWE scores for each participant across stimulation types, not controlling for session effects. (D) Changes in SWE scores relative to sham stimulation; the horizontal line indicates the mean change score. * *p* < 0.05.

SWE scores were lower after cathodal stimulation compared to sham stimulation (beta = −3.95, *t*(46) = −2.40, *p*_uncorrected_ = 0.02, *d* = −0.36; Figure 4A–C). This effect did not survive correction for multiple comparisons using the Benjamini-Hochberg procedure (*p*_corrected_ = 0.08). SWE scores did not significantly differ after anodal stimulation compared to sham stimulation (beta = −1.64, *t*(46) = −1.00, *p*_uncorrected_ = 0.32, *d* = −0.15) or after anodal stimulation compared to cathodal stimulation (beta = −2.31, *t*(46) = −1.40, *p*_uncorrected_ = 0.17, *d* = −0.21). The relative within-subject change in scores (the difference between sham and each active stimulation condition for each participant) were also not significantly different from each other (*t*(46) = −0.88, *p*_uncorrected_ = 0.38; Figure 4D). TDCS did not have significant effects on the PDE or RAN tasks (*p* > 0.05; Figure S1).

LME models also showed effects across sessions. SWE scores decreased between sessions 1 and 3 (beta = −3.93, *t*(46) = −2.40, *p* = 0.02, *d* = −0.36; Figure 4A) while RAN scores improved between sessions 1 and 3, indicating a practice effect (beta = 1.89, *t*(46) = 2.09, *p* = 0.04, *d* = 0.20). Neither of these effects survived after controlling for multiple comparisons with the Benjamini-Hochberg procedure (*p*_corrected_ = 0.08, *p*_corrected_ = 0.14, respectively). No effects were seen between sessions 1 and 2 (*p* > 0.05; Figure S1).

#### Effect of cerebellar tDCS on reading accuracy

Backward stepwise regression removed the interaction term (tDCS × Session) in the single-word reading accuracy model, resulting in a reduced model with only tDCS and session. Residuals for the WRAT5 Word Reading LME models were not normally distributed, therefore the log of WRAT5 scores was used in the model to maintain a normal distribution. Consistent with our hypotheses, tDCS did not have an effect on single-word reading accuracy (*p* > 0.05; Figure S2). Marginal evidence of a session effect was seen between sessions 1 and 2 with a slight decrease in performance (beta = −0.02, *t*(46) = −1.97, *p* = 0.05, *d* = −0.28), but no change was evident between sessions 1 and 3 (*p* > 0.05). This marginal effect did not survive corrections for multiple comparisons with the Benjamini-Hochberg procedure (*p*_corrected_ = 0.16).

#### Effect of cerebellar tDCS on general processing speed

Backward stepwise regression removed the interaction term (tDCS × Session) in all processing speed models, resulting in reduced models including only tDCS and session. The effect of tDCS was not statistically significant. Symbol Search showed practice effects through session 2 (beta = 2.66, *t*(46) = 6.97, *p* < 0.001, *d* = 0.84) and session 3 (beta = 3.62, *t*(46) = 9.48, *p* < 0.001, *d* = 1.14; Figure S3). These effects survived corrections for multiple comparisons with the Benjamini-Hochberg procedure (*p*_corrected_ < 0.001). Coding showed practice effects through session 2 (beta = 0.89, *t*(46) = 3.45, *p* = 0.001, *d* = 0.26) and session 3 (beta = 1.37, *t*(46) = 5.29, *p* = 0.001, *d* = 0.41; Figure S4), which survived corrections for multiple comparisons (*p*_corrected_ = 0.006, *p*_corrected_ < 0.001, respectively).

### Results Summary

Cathodal cerebellar tDCS disrupted timed single-word reading as measured by the TOWRE-2 SWE scores. Cerebellar tDCS did not affect timed nonword reading, RAN, single-word or nonword reading accuracy, or general processing speed.

## DISCUSSION

In the current study, we investigated how cerebellar neuromodulation impacted performance on various types of reading and processing speed measures in young adult readers. We hypothesized that (1) modulating activity in the right posterolateral cerebellum would affect timed single-word reading fluency scores but would not affect untimed single-word reading accuracy measures and (2) anodal tDCS would improve reading fluency while cathodal tDCS would disrupt reading fluency. Our hypotheses were partially supported by the finding that cathodal tDCS disrupted SWE scores, a timed single-word reading fluency measure, but active tDCS did not influence other timed reading or processing speed measures. Results also suggested that at 2 mA of current, anodal and cathodal stimulation shifted scores mostly in the same direction rather than having consistent opposing effects. Additionally, active stimulation, whether anodal or cathodal, did not impact WRAT5 Word Reading, a single-word reading accuracy measure. Furthermore, we provided evidence that the right posterolateral cerebellum worked in a domain-specific manner, as the impact of cerebellar tDCS was limited to reading fluency measures and did not significantly affect rapid automatized naming or general processing speed scores.

These findings are in line with previous research investigating the cerebellum’s involvement in reading and automating cognitive skills. The current study found that cathodal stimulation of the right posterolateral cerebellum disrupted real word reading fluency. Reading fluency is achieved when reading is fast and automatic (Snowling et al., 2020), and Sokolov et al. (2017) suggested that the cerebellum may aid the cortex in automating cognitive processes by coordinating the cortical regions within a network to be primed for certain input, which would increase speed of output. This may be what is occurring during fluent reading in young adults with no history of reading difficulties. Cathodal (inhibitory) stimulation could disrupt the output from the right posterolateral cerebellum to the cortex, which may then negatively impact the management of the cortical reading network. Specifically, communication between the cerebellum and left IFG may be altered in a way that affects reading fluency. Supporting this, Rice et al. (2021) showed that anodal right posterolateral cerebellar tDCS altered functional activation in the left IFG compared to sham stimulation.

We did not find significant differences in reading fluency between anodal and cathodal tDCS, which goes against the prediction that anodal and cathodal stimulation would have opposing effects on behavior. That said, anodal and cathodal tDCS do not consistently show opposite effects. This inconsistency may be due to many factors, including target and reference electrode placement, current intensity, and current duration (van Dun et al., 2016). Additionally, at higher intensities, the directionality of electrical signaling can reverse, as seen in motor evoked potentials following 2 mA of current compared to 1 mA (Krause & Cohen Kadosh, 2014). This may be why in the current study, which utilized 2 mA of current, instead of anodal stimulation increasing reading fluency scores and cathodal stimulation decreasing reading fluency scores, we found that both anodal and cathodal tDCS disrupted reading fluency (although the anodal effect did not reach statistical significance).

Consistent with our hypothesis, we did not find evidence that cerebellar neuromodulation affected reading accuracy, which, in the current study, was measured with an untimed single-word reading task (WRAT5 Word Reading). This finding is consistent with Norton et al.’s (2014) study investigating neural correlates of phonological awareness and rapid naming skills in children with typical reading development and DD (defined by the double-deficit model) in either phonology, rapid naming, or both. The phonological awareness tasks did not emphasize speed, whereas the rapid naming tasks did. Activity in the right posterolateral cerebellum (lobule VI) was associated with rapid naming scores but not with phonological awareness scores. Additionally, a more recent study by Ang et al. (2020) found similar results, showing a connection between cerebellar lobule VI activity at rest and rapid naming skills but not untimed phonological awareness skills. The posterolateral cerebellum does appear in reading neuroimaging meta-analyses utilizing studies of untimed single word reading (Turkeltaub et al., 2002) or reading-related tasks ranging from rhyme judgment to reading whole stories (Martin et al., 2015). That said, Ang et al. (2020) reported that resting state activation in right lobule VI correlated with phonological awareness, but this relationship disappeared when rapid naming skills were included as a covariate and regressed out of the model, suggesting that the cerebellum primarily supports the automaticity and speed of reading-related skills. This may also be applicable to previous meta-analyses that did not take into account the covariance of phonological and rapid naming skills in the analysis.

There are mixed findings regarding the cerebellum’s role in general processing speed. Some studies find a relationship between the cerebellum and processing speed measures (Forn et al., 2009; Razlighi et al., 2017; Wong et al., 2021), but this is not consistent (Habeck et al., 2016; Sweet et al., 2005). For example, two similar studies found conflicting cerebellar results (Habeck et al., 2016; Razlighi et al., 2017). Both studies used the same three processing speed tasks (digit span, pattern comparison, letter comparison) to create a singular cognitive processing speed variable, yet one reported cerebellar involvement in processing speed and the other did not, making interpretation difficult. There are even conflicting results within processing speed measures: an fMRI-compatible Coding measure found right cerebellar hemisphere activation (Forn et al., 2009; called Symbol Digits Modality Test in the publication but is the same administration and task as Coding), but an fMRI-compatible Symbol Search task did not engage the cerebellum (Sweet et al., 2005). It is possible that the numeric stimuli used in Coding could recruit the cerebellum in a way that the uncommon symbolic stimuli used in Symbol Search do not, and that the parameters of the tasks defined these results. We did not find cerebellar neuromodulation to influence either Coding or Symbol Search scores, leaving this an open question for future investigation. While our results point to null effects of cerebellar neuromodulation on processing speed measures, we also note that our sample was small and there may be power limitations. Further, our tDCS montage may not have been ideally targeting regions involved in general processing speed measures (e.g., bilateral lobule VI; Forn et al., 2009).

Our correlation results are in line with previous studies showing a significant relationship between RAN and reading fluency (Norton & Wolf, 2012; Wolf & Bowers, 1999). We report statistically significant correlations between RAN and both TOWRE-2 reading fluency measures; in particular, there was a strong correlation between RAN and SWE, which requires automatic reading of real single words. But it is important to discuss the lack of statistically significant correlations between reading fluency measures and processing speed measures in the current study, given previous studies have reported this relationship (Gerst et al., 2021; Jacobson et al., 2011; Norton & Wolf, 2012; Rucklidge & Tannock, 2002; Wolf & Bowers, 1999). None of the reading fluency or RAN measures strongly correlated with either of the processing speed measures. We did, though, find a statistically significant positive correlation between an untimed word reading accuracy measure taken before tDCS application and Symbol Search (*r* = 0.44) and a comparable, albeit nonsignificant, correlation with Coding (*r* = 0.38). This is consistent with a line of work showing that processing speed scores were related to untimed single-word reading, even after controlling for naming speed (RAN; Christopher et al., 2012; McGrath et al., 2011; Peterson et al., 2017; Shanahan et al., 2006). Reading fluency and general processing speed are moderately linked in the literature, so it is surprising that these measures were not correlated in this sample. Reading fluency and general processing speed tasks could measure processes occurring at different timings and levels of the neural networks involved in reading (i.e., general processing speed ability may rely more on domain-general processes like attention, scanning, sequencing, and working memory) whereas rapid, automatic reading may recruit later occurring, higher-order cognitive processes (Norton & Wolf, 2012). This may account for why reading fluency measures were not highly correlated with general processing speed measures in this cohort.

### Limitations

The current study used tDCS to directly manipulate cerebellar activity to measure the specific role of the cerebellum during single-word reading, rapid naming, and processing speed tasks. In the reading literature, tDCS has most frequently been applied to the cerebral cortex, with the most consistent impacts being improved reading efficiency following anodal tDCS targeting the left temporoparietal area in individuals diagnosed with DD (Cancer & Antonietti, 2018). When targeting the cerebellum with tDCS, many studies have placed the reference electrode on the buccinator muscle (Ferrucci et al., 2015), whereas the current study used the ipsilateral upper arm. This may have affected the focality of the current, as our configuration had a larger distance between target and reference electrode (Klaus & Schutter, 2021). This, in turn, may have limited the specificity of the regional electrical current. With anatomical and functional maps of the cerebellum indicating small subregions of the cerebellum engaged during many types of cognitive tasks (e.g., King et al., 2019), our montage might have overlapped with multiple anatomical and/or functional regions that were not directly associated with language and/or reading skills.

Another potential limitation could stem from practice effects. Participants were administered the same tasks, albeit not in the same order, in all three sessions. Some of these measures had alternate forms (e.g., SWE and PDE have forms A, B, C, D; WRAT5 Word Reading has forms blue, green) which were pseudorandomized within participants. However, because not all of the measures had alternate forms, for some tasks the same stimuli were viewed in each session (e.g., RAN, processing speed measures). We accounted for potential practice effects by including session number in the LME models while investigating our variable of interest: tDCS. Therefore, any effects of tDCS that were found, such as in SWE, should not be confounded by multiple exposures. Moreover, the significant effect of cathodal stimulation on SWE in particular should not be due to practice effects, because this measure had four alternate forms. While participants may have become more comfortable with the task over time, they did not see the same words or pseudowords each time. Counterbalancing the order of tDCS conditions across participants should also minimize the impact of practice effects on the results. That said, it is possible that practice effects for measures without alternate forms may have contributed additional measurement error, making it difficult to statistically identify variance due to tDCS and resulting in false negative effects for some tasks.

It is also important to note that tDCS has a relatively small effect size (Minarik et al., 2016), and it is possible that our sample size was not large enough to detect a small effect of tDCS on other measures. In the current study, we found a statistically significant effect of cathodal tDCS on single-word reading fluency scores, though the effect size was relatively small (*d* = −0.36). While small, this effect size is consistent with typical effect sizes found in psychology studies (Brysbaert & Stevens, 2018). Effect sizes for other reading tasks that did not reach statistical significance were small to nearly nonexistent (see Table S3), indicating that power was not the primary issue driving null results on other measures. In interpreting our null results, it is also important to consider sampling characteristics. TDCS may not have as large an impact in individuals with typical reading development who might have optimal network efficiency that is unperturbed by neuromodulation (Horvath et al., 2015). Larger effect sizes of tDCS may be seen in individuals with DD, where neuromodulation may have a greater impact on network efficiency (e.g., Turkeltaub et al., 2012).

Finally, it is evident from the data that not all individuals in the study showed the same effect of cerebellar tDCS; most of the participants showed a disruption in fluency after active cerebellar tDCS while a small subset of participants showed improved fluency relative to their post-sham tDCS (control) score. This individual variability is a known phenomenon in neuromodulation studies (see Krause & Cohen Kadosh, 2014) and has obvious implications for potential translation to clinical populations. Future studies will need to combine neuromodulation with neuroimaging in order to evaluate whether factors such as initial brain state or task activation patterns predict an individual’s response to cerebellar tDCS.

### Conclusions and Future Directions

Cathodal stimulation of the cerebellum disrupted single real word reading fluency but did not affect untimed reading accuracy in a group of young adults with typical reading development. Cerebellar neuromodulation also did not affect RAN or general processing speed scores, suggesting the right posterolateral cerebellar contribution to reading is relatively specific to reading fluency. These findings support the hypothesis that the cerebellum is involved in reading fluency in typical readers. Although the current study resulted in decreased reading fluency after modulating cerebellar activity, this may be because neuromodulation disrupted optimally functioning, efficient reading networks in typical young adult readers. Therefore, future studies will need to investigate the impact of cerebellar neuromodulation on reading speed in individuals with reading fluency difficulties.

## ACKNOWLEDGMENTS

We thank the past and current members of the American University Developmental Neuroscience Lab: Emily Barnes, Sheila Carr, Sydney Cerveny, Joe Dust, Alexia Hyde, Alexandra Kauffman, Alyssa Miller, Laura Rice, Hayli Spence, Alicia Stringham, and Honeyeh Younesie. This work was supported by funding from American University to Marissa M. Lee and Catherine J. Stoodley.

## FUNDING INFORMATION

Marissa M. Lee, American University Graduate Student Research Award. Lauren M. McGrath, National Institutes of Health (https://dx.doi.org/10.13039/100000002), Award ID: P50HD027802. Lauren M. McGrath, National Institutes of Health (https://dx.doi.org/10.13039/100000002), Award ID: R15HD086662. Catherine J. Stoodley, American University Faculty Mellon Research Award.

## AUTHOR CONTRIBUTIONS

**Marissa M. Lee**: Conceptualization: Equal; Data curation: Lead; Formal analysis: Lead; Investigation: Lead; Visualization: Lead; Writing – original draft: Lead; Writing – review & editing: Equal. **Lauren M. McGrath**: Conceptualization: Supporting; Formal analysis: Supporting; Methodology: Supporting; Writing – review & editing: Supporting. **Catherine J. Stoodley**: Conceptualization: Lead; Formal analysis: Supporting; Investigation: Supporting; Supervision: Lead; Writing – review & editing: Equal.

## COMPETING INTERESTS

The authors declare the following, which may be considered a potential competing interest: Lauren M. McGrath receives royalties from the textbook *Diagnosing Learning Disorders: From Science Into Practice* (3rd edition) from Guilford Press.

## DATA AND CODE AVAILABILITY STATEMENT

Data are available via https://github.com/stoodley/NoL2023.

## Supplementary Material



## TECHNICAL TERMS

Functional MRI: An indirect measure of brain activity.
Structural imaging: Measures brain anatomy or an aspect of brain structure, such as regional volume or cortical thickness.
Decoding: The ability to use letter–sound relationships to read a word.
Reading fluency: The ability to read quickly and accurately while also maintaining comprehension.
Meta-analyses: Statistically combine multiple similar studies to determine how research findings converge.
Orthographic depth: A measure of how consistently letters map to sounds in a language.
Automatization: Refers to when a skill can be completed automatically.
Functional connectivity: A statistical measure of how signal changes correlate over time; thought to reflect functional brain networks.
Double-deficit model: Proposes that phonological processing and naming speed/fluency independently contribute to reading difficulties.
Neuromodulation: A technique where neuronal activity is altered, usually by electrical, magnetic, or pharmaceutical methods.
