# Supplementary material for: Cerebellar Neuromodulation Impacts Reading Fluency in Young Adults
Source: Neurobiol Lang (Camb). 2024 Aug 15;5(3):736–56. doi: 10.1162/nol_a_00124 (PMC11338301; doi:10.1162/nol_a_00124)

Table S1. Reported symptoms during and after neuromodulation

Symptom	Sham	Anodal	Cathodal
**During tDCS**			
Burning	1.02±1.98 [0-7]	0.94±1.61 [0-6]	1.40±2.12 [0-7]
Concentrating	0.28±0.63 [0-2]	0.58±0.76 [0-3]	0.62±1.12 [0-4]
Fatigue	0.52±0.81 [0-2.5]	0.24±0.60 [0-2]	0.28 ±0.52 [0-2]
Headache	0.32±0.98 [0-4.5]	0.16±0.47 [0-2]	0.22±0.48 [0-2]
Itching	1.54±2.13 [0-7]	3.64±2.44 [0-7]	1.78±2.03 [0-7]
Mood Change	0.02±0.10 [0-0.5]	0.04±0.20 [0-1]	0.10±0.32 [0-1.5]
Nervousness	0.22±0.58 [0-2]	0.52±1.26 [0-5]	0.42±0.94 [0-4]
Pain	0.50±1.50 [0-7]	0.32±0.84 [0-4]	0.78±1.32 [0-5]
Tingling	1.76±1.71 [0-6]	2.18±1.67 [0-7]	2.54±1.97 [0-7]
Visual Perception	0.06±0.30 [0-1.5]	0.16±0.47 [0-2]	0.06±0.17 [0-0.5]
Visual Sensation	0	0	0.16±0.49 [0-2]
**After tDCS**			
Burning	0.04±0.20 [0-1]	0.04±0.20 [0-1]	0.08±0.24 [0-1]
Concentrating	0.18±0.52 [0-2]	0.26±0.69 [0-3]	0.36±0.85 [0-2.5]
Fatigue	0.42±0.86 [0-3]	0.20±0.50 [0-2]	0.48±0.99 [0-4.5]
Headache	0.30±1.15 [0-5.5]	0.38±1.12 [0-5.5]	0.12±0.30 [0-1]
Itching	0.18±0.72 [0-3.5]	0.60±1.15 [0-5]	0.48±0.97 [0-4]
Mood Change	0	0	0.06±0.30 [0-1.5]
Nervousness	0	0	0.10±0.41 [0-2]
Pain	0.04±0.20 [0-1]	0	0.02±0.10 [0-0.5]
Tingling	0	0.12±0.44 [0-2]	0.26±0.54 [0-2]
Visual Perception/ Sensation	0.04±0.20 [0-1]	0	0.02±0.10 [0-0.5]
Visual Sensation*	-	-	-

*combined with visual perception

Table S2. Uncorrected and corrected p-values.
The Analysis column indicates the task (e.g., swe = sight word efficiency) and independent variable (e.g., A = anodal tDCS) for the linear mixed effects models. Sham tDCS was used as a reference for Anodal (A) and Cathodal (C) stimulation. Session 1 was used as a reference for Session 2 (2) and Session 3 (3). P-values were corrected using the Benjamini-Hochberg procedure.

Analysis	Pval (uncorrected)	Pval (corrected; Benjamini-Hochberg)
swe_A	0.323	0.482823529
swe_C	0.02	0.084
swe_2	0.172	0.3408
swe_3	0.021	0.084
pde_A	0.199	0.3408
pde_C	0.392	0.495157895
pde_2	0.153	0.3408
pde_3	0.213	0.3408
ran_A	0.427	0.5124
ran_C	0.504	0.576
ran_2	0.805	0.805
ran_3	0.042	0.144
wrat_A	0.153	0.3408
wrat_C	0.342	0.482823529
wrat_2	0.054	0.162
wrat_3	0.622	0.649043478
coding_A	0.604	0.649043478
coding_C	0.366	0.488
coding_2	0.001	0.006
coding_3	3.00E-06	2.40E-05
ss_A	0.168	0.3408
ss_C	0.191	0.3408
ss_2	1.00E-08	1.20E-07
ss_3	2.00E-12	4.80E-11

Table S3. Effect sizes.
Model indicates the dependent variable of interest. Variable indicates the independent variable in the linear mixed effects model. Sham tDCS was used as a reference for Anodal and Cathodal tDCS. Session 1 was used as a reference for Session 2 (ses2) and Session 3 (ses3). Betas are the standardized beta values from the linear mixed effects model output (RStudio, Version 4.1.2, jtools package). Effect sizes were calculated based on the equation from Brysbaert and Stevens (2017).

Model	Variable	Beta	Effect Size
SWE	anodal	-1.64	-0.1502628
	cathodal	-3.95	-0.3619135
	ses2	-2.28	-0.208902
	ses3	-3.93	-0.360081
PDE	anodal	0.02	0.25819889
	cathodal	0.01	0.12909944
	ses2	0.02	0.25819889
	ses3	0.02	0.25819889
RAN	anodal	0.72	0.07560123
	cathodal	-0.61	-0.064051
	ses2	0.22	0.02310038
	ses3	1.89	0.19845322
WRAT	anodal	0.02	0.28284271
	cathodal	0.01	0.14142136
	ses2	-0.02	-0.2828427
	ses3	0.01	0.14142136
Coding	anodal	-0.13	-0.0387069
	cathodal	-0.24	-0.071459
	ses2	0.89	0.26499364
	ses3	1.37	0.40791156
SS	anodal	0.54	0.17076299
	cathodal	-0.51	-0.1612762
	ses2	2.66	0.84116586
	ses3	3.62	1.14474451
SWE	anodal/cathodal comparison	-2.31	-0.2116507

Figure S1. tDCS did not have an effect on PDE (log) or RAN. Significant differences in RAN scores were seen between sessions 1 and 3.

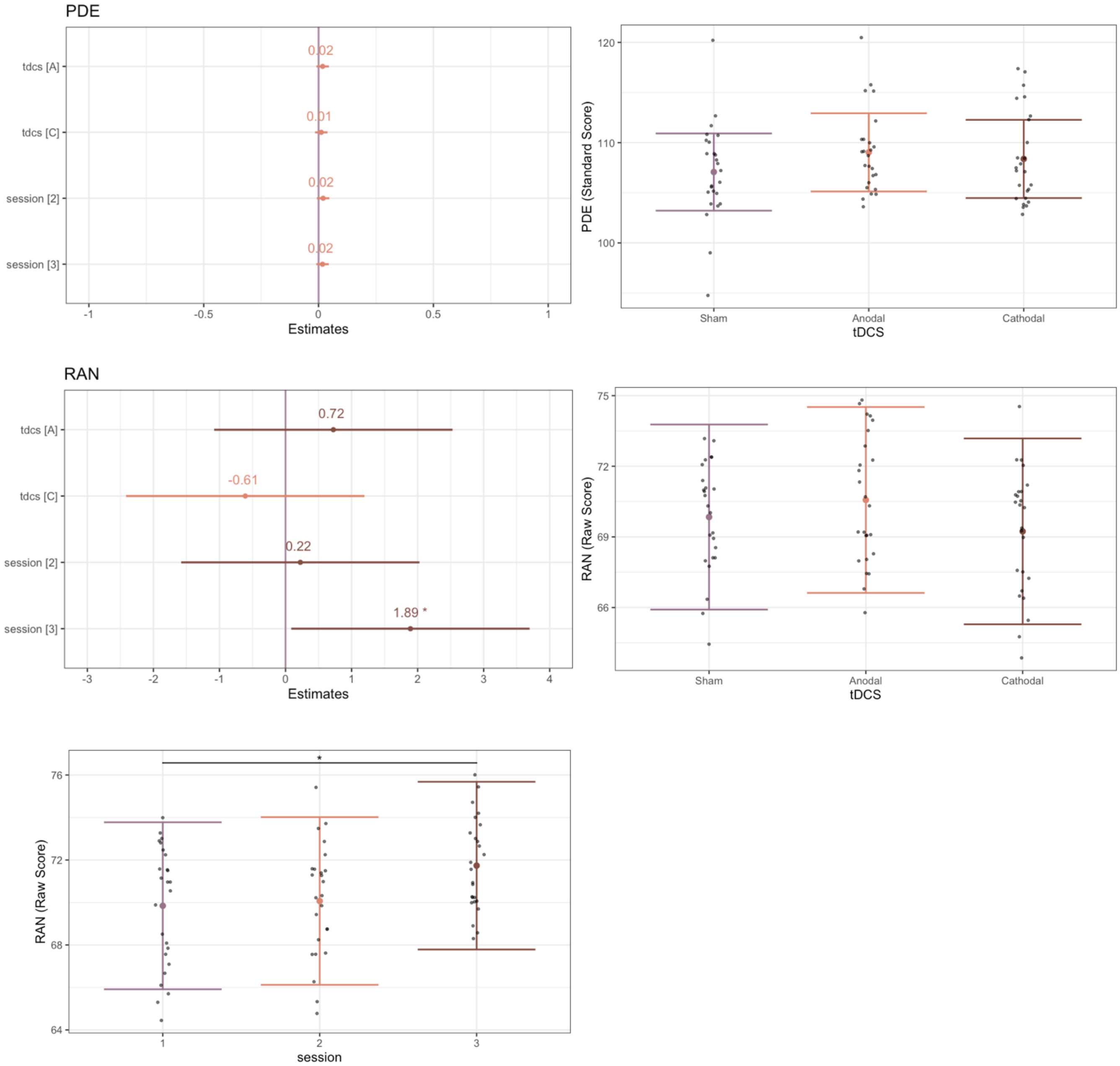

Figure S2. tDCS did not have an effect on WRAT5 Word Reading scores (log). WRAT5 scores were marginally reduced from session 1 to session 2 (p = 0.054) but were not significantly different in session 3.

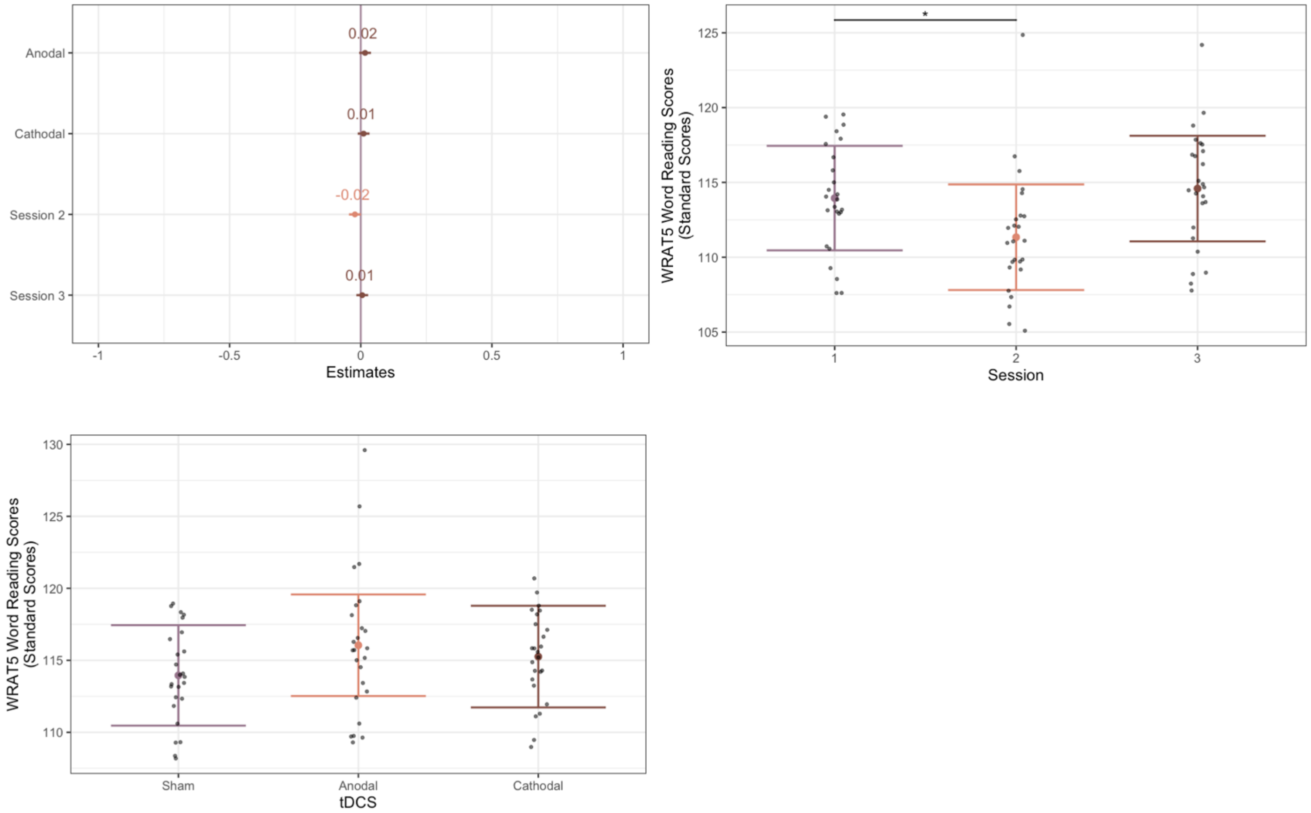

Figure S3. No effects of tDCS on Symbol Search scores. Symbol Search scores improved from sessions 1 to sessions 2 and 3 ($p < 0.001$).

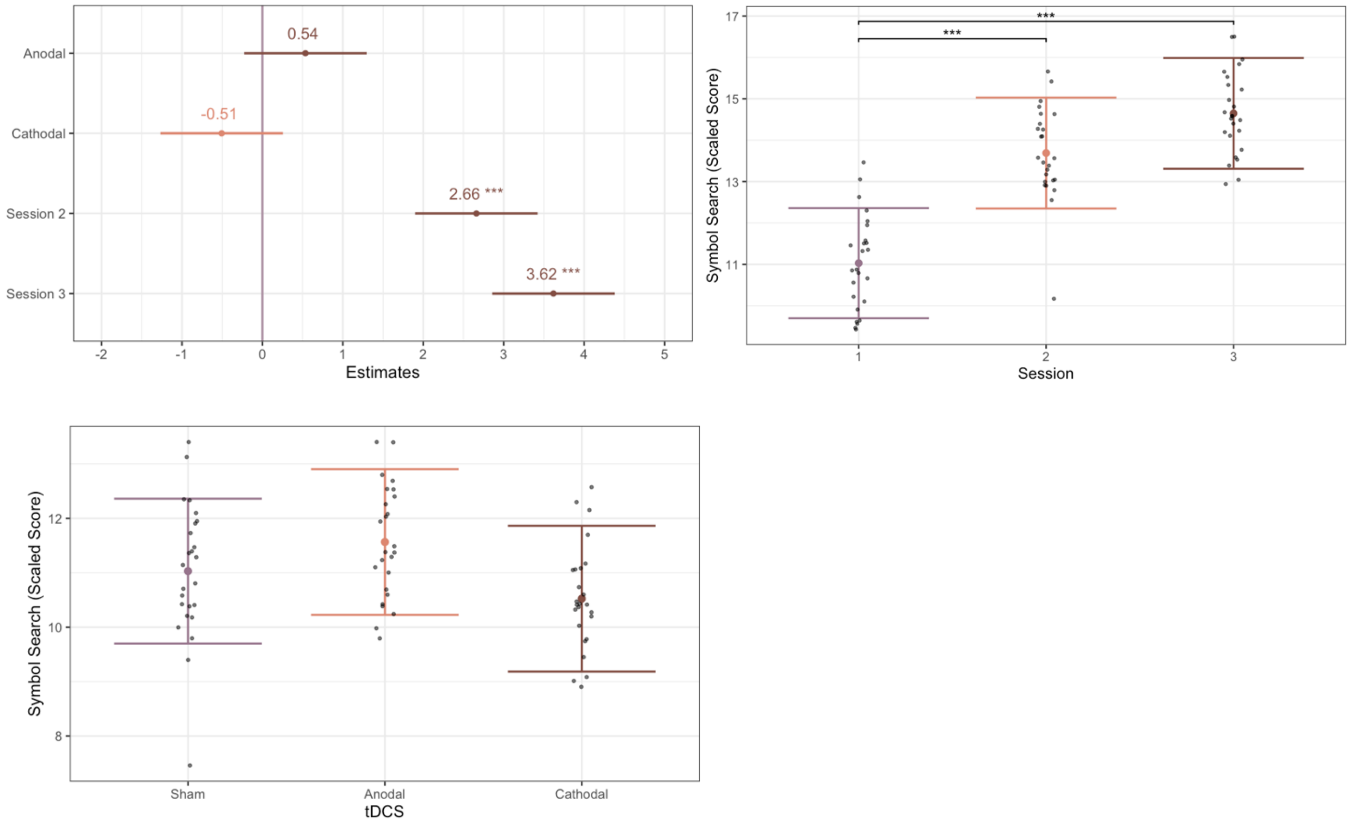

Figure S4. No effects of tDCS on Coding scores. Coding scores improved from sessions 1 to sessions 2 and 3 ($p < 0.001$).

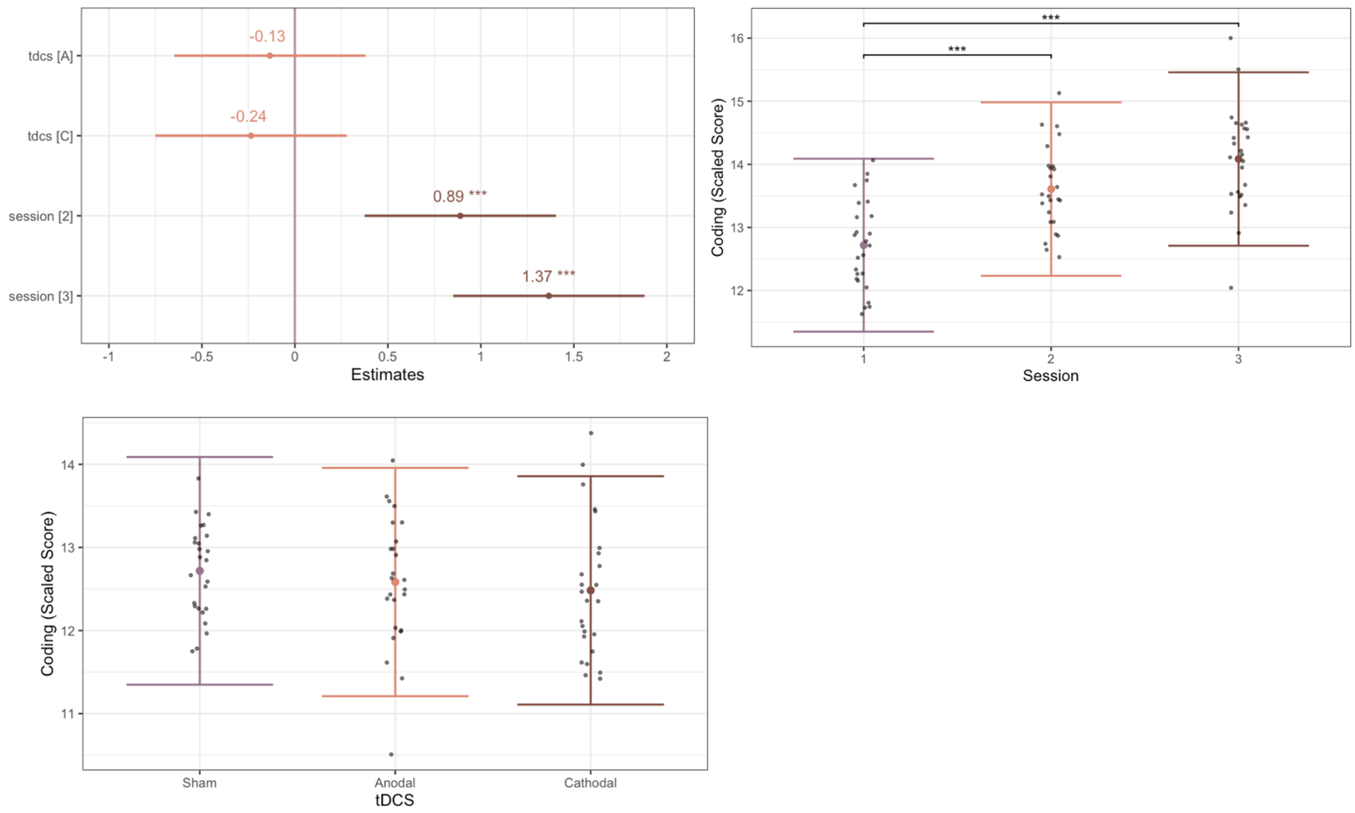

Supplement: Supplementary file 1 [file nol-5-3-736-s001.pdf]
